# Exosome-mediated delivery of microRNAs by root-knot nematodes

**DOI:** 10.1371/journal.ppat.1013908

**Published:** 2026-02-09

**Authors:** M. Willow H. Maxwell, Alex Papp, Bharat Rohilla, Caitlin Simpson, Martin Fuller, Suruchi Roychoudhry, Chris A. Bell

**Affiliations:** Faculty of Biological Sciences, University of Leeds, Leeds, United Kingdom; The Ohio State University, UNITED STATES OF AMERICA

## Abstract

Plant-parasitic nematodes secrete molecules to manipulate their hosts, but little is known about their mode of delivery and packaging. Here, we describe microRNA-containing exosomes that are secreted by root-knot nematodes and systemically increase host susceptibility. By revealing a novel mode of nematode-plant communication, our findings outline a mechanism for the delivery of nematode patho-molecules, offering a new target for disrupting parasitism at the level of vesicle-mediated delivery.

## Introduction

**Fig 1:**
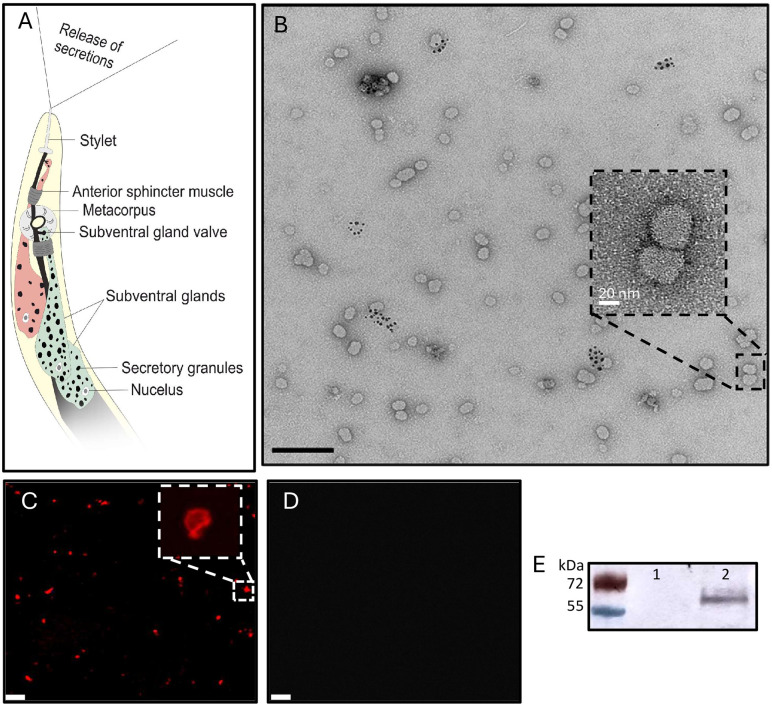
Characterisation of exosomes secreted by the plant-parasitic nematode *Meloidogyne incognita.* **A)** Schematic representation of the subventral gland secretion pathway of a second-stage juvenile. **B)** Transmission electron microscopy of resorcinol-induced secretions at 30k magnification, scale bar represents 200 nm. Inset is at 150k magnification, scale bar represents 20 nm. **C)** Exosomes treated with CellMask Deep Red and visualised using confocal microscopy. Scale bar represents 200 nm. **D)** As in C, but with 10% Triton X-100 treatment prior to confocal microscopy. **E)** Western blot of the tetraspanin CD63 from non-induced (1) and resorcinol-induced secretions (2).

Plant-parasitic nematodes pose a persistent and significant threat to agriculture. Among them, the root-knot nematode *Meloidogyne incognita* is widely regarded as the most destructive [1], owing to its extremely broad host range of over 4,000 plant species [2]. Root entry and the establishment of a complex feeding site within the host is facilitated by a suite of nematode-secreted “patho-molecules”. The secretions of second-stage juvenile (J2) nematodes are arguably the most studied and originate from two subventral gland cells (Fig 1A), which are active pre-invasion and contribute to the degradation of the plant cell wall and suppression of host immune responses [3–7]. Gland cell products are stored in Golgi-derived granules (700–1100 nm diameter), prior to their release [8]. Each granule contains “minute spherical vesicles” [8], with their biogenesis, structure and function remaining unknown. Over the past few decades, researchers have thoroughly detailed the repertoire and role of secreted effector proteins [9], however, we have little insight into other secreted molecules, such as nucleic acids, or whether these cargo are “naked” or packaged to aid protection/trafficking. Small RNA-containing exosomes are being increasingly identified in other organisms, such as animal parasitic nematodes [10,11], and there may be parallels in plant systems.

## Results and discussion

Subventral gland secretions are naturally induced by root exudates, however resorcinol, a neurotransmitter, triggers their release *in vitro* [5]. Transmission electron microscopy of concentrated secretions revealed exosomes approximately 25–70 nm in diameter (Fig 1B; mean 49.7 nm, SEM 0.9 nm, n = 512), up to 50% smaller than vesicular secretions by other nematode species [10,11]. No exosomes were found in non-induced solutions, or the resorcinol stock.

We investigated the structure of nematode secreted exosomes for the long-term objective of targeting their integrity, therefore potentially disrupting an entire patho-molecule delivery system. CellMask staining inferred that secreted structures were enclosed in lipid membranes (Fig 1C), which commonly encapsulate cargo transported *vice versa* between pathogens and their hosts [12]. This is supported by exosome disruption via detergent trea(Fig 1D). Exosome lipid bilayers are abundant with tetraspanins that enable membrane curvature, selection of exosome cargo and the direction and adhesion of exosomes to cell membranes [10,18,19]. Tetraspanin CD63, an exosome marker [13], was identified within the secreted exosome fraction (Fig 1E), consistent with underpinning genes identified within the nematode genome (Minc3s00247g08503 & Minc3s01356g23074). Together this infers the secretion of lipid and protein containing structures, likely exosomes.

**Fig 2:**
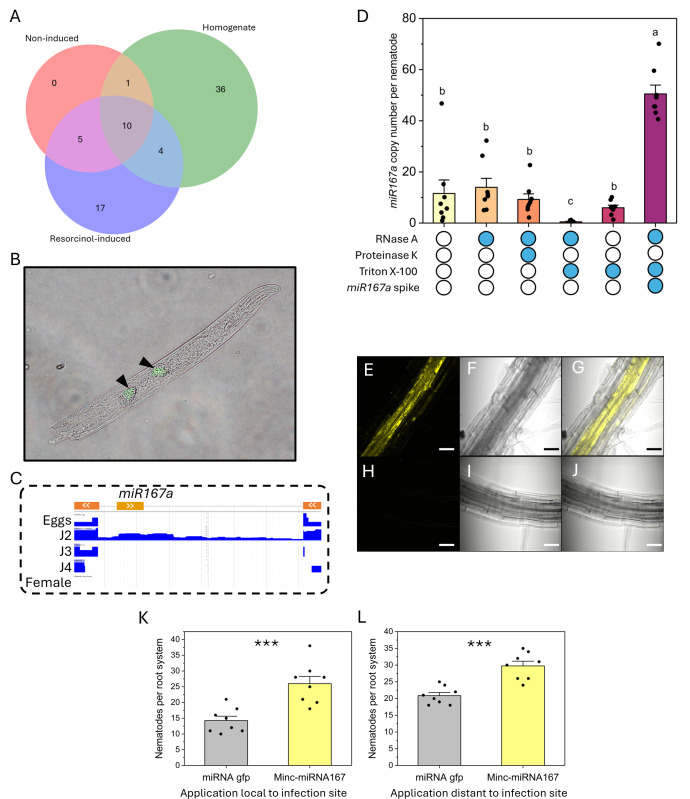
Characterisation of a miRNA present within plant-parasitic nematode secreted exosomes. **A)** The number of miRNA with expression counts >10 identified from sequencing of *M. incognita* (RKN) homogenate, resorcinol-induced secretions, and non-induced solutions. **B)** Overlay images of an *in situ* hybridisation chain reaction of *minc-miR167a* on a second-stage *M. incognita* juvenile. Subventral glands are indicated with black arrows. **C)** Intronic and reverse strand location of *minc-miR167a* within the *M. incognita* genome. The outer gene exons are represented on the top row in orange, whilst *minc-mi167a* is in yellow. Subsequent rows indicate the expression levels of each genomic region, with J2s (second-stage juveniles) uniquely expressing the intron containing *minc-mi167a*. **D)** RNA protection assay to determine the localisation of *minc-miR167*. The *minc-miR167a* copy number within resorcinol-induced secretions was estimated using Taqman probe qPCR. Secretions were subjected to combinations of Protienase K, Triton X-100 and RNase A treatments (indicated on x-axis) prior to RNA purification to infer the localisation of *minc-miR167a* within protein or lipid shells. On samples that were depleted in *minc-miR167a,* synthesised RNA were spiked in post-RNA extraction to assess the impact of Triton X-100 and RNase A treatments on Taqman-based detection methodology. N = 8; P < 0.05 One-way ANOVA Tukey post-hoc analyses. **E, F & G)** Visualisation of cy3-tagged *minc-miR167a* within the root vasculature of *Arabidopsis thaliana* Col-0, via confocal microscopy **(E)**, with brightfield (F) and overlay **(G)**. **H, I & J)** Mock treated *A. thaliana* Col-0 roots visualised by confocal microscopy. Scale bars represents 40 µm. K & L) 20 nM untagged ds*minc-miR167a* was applied to *M. incognita* infected root tips **(J)**, or root tips distant to infection sites **(K)**, every day for four days before infection was quantified. Asterisk denote significance at P < 0.001 Two-sample t-test.

Given that exosomes from other pathogens/parasites contain nucleic acids [11,14], we investigated whether this is also true for *M. incognita.* Bioinformatic studies have mapped the miRNAome of a different plant-parasitic nematode species [15], however we are still unsure whether miRNA are secreted and if so, whether they are within exosomes. Small RNA sequencing of *M. incognita* secreted exosomes revealed 17 miRNA that were enriched within resorcinol-induced secretions compared to uninduced and homogenised nematodes (Fig 2A). Lack of these miRNA within the homogenate is potentially due to an exhausted secretion in the presence of resorcinol. The most abundant miRNA enriched in secretions was *Mi-miR167a*, which is widespread across plant species and regulates auxin signalling pathways that are integral to root formation and systemic resistance [16]. *M. incognita* infection represses the general plant *miR167* family at 7 & 14 dpi [17], however plant *miR167a* is highly abundant during early parasitism [18], coinciding with the activity of the subventral glands, evidencing the dynamic and context-dependent role of miRNAs, as seen in other systems where plant *miR167a/d* negatively/positively regulate immunity to fungal [19,20] and bacterial infection [21]. The high expression of *Mi*-*miR167a* in secretions, whilst absent within non-induced samples, led to further analyses. *In situ* hybridisation of *Mi-miR167a* confirmed its nematode origin and expression within J2 subventral gland cells (Fig 2B). Genome analysis revealed that *Mi-miR167a* is located within an intron, oriented antisense to an adjacent gene of unknown function (Fig 2C) and has 100% sequence homology throughout the *M. incognita* species group. Although the neighbouring gene has orthologues in cyst nematodes, *mi167a* sequence was not identified. This intron appears to be only expressed in J2s (Fig 2C), consistent with the identification of *Mi-miR167a* within their secretions and the reported negative impact of plant-*miR167* later during nematode development [17].

An RNA protection assay was performed to determine whether miRNA were internal to the observed exosomes. Taqman-probe qPCR estimated approximately 11.6 *Mi-miR167a* copies per nematode obtained from the exosome fraction of resorcinol secretions (Fig 2D). Treatment with Proteinase K and RNase A did not affect *Mi-miR167a* detection, whereas Triton X-100 followed by RNase A caused a marked reduction (approx. 0.47 copies detected per nematode). Triton X-100 alone had no effect on *Mi-miR167a* detection, and *Mi-miR167a* spiked into Triton X-100 + RNase A-treated samples post-RNA extraction remained detectable, confirming that detergent treatment did not interfere with the detection assay. Together, these results indicate that *Mi-miR167a* is protected within detergent-sensitive vesicles, consistent with exosomes, rather than free-RNA or within protein-RNA complexes.

To determine the impact of *Mi-miR167a* within the plant, we applied synthetic Cy3-tagged *Mi-miR167a* to *Arabidopsis thaliana* roots. Systemic translocation of Cy3-*Mi-miR167a* was observed via the root vasculature (Fig 2E), a route known to transport pathogen-derived miRNAs [22], suggesting that inter-kingdom miRNAs translocate through a general plant transportation pathway. Application of untagged-*Mi-miR167a* to infection sites increased *M. incognita* infection of tomato roots compared to *gfp*-targeting miRNA application (Fig 2K). *Mi-miR167a* application at uninfected root tips distant from the nematode infection site also increased invasion of *M. incognita,* supporting a systemic effect (Fig 2L), via vascular tissue.

In summary, we identify and characterise a potential exosome-based subventral gland secretion system of the root-knot nematode. We propose that secreted miRNA, and potentially certain effector proteins if consistent with other patho-systems, are released in exosomes, which likely protect the biological cargo *en route* to the host, potentially enabling their delivery into host cells. Our findings establish a foundation for targeting the patho-molecule delivery pathway itself, offering a broader strategy to disrupt parasitic interactions beyond single molecule approaches.

## Methods

### Induction of nematode secretions

*Meloidogyne incognita* (VW6) were maintained on tomato plants (‘Ailsa Craig’) at 25°C. Infected roots were placed in a misting chamber where high humidity encouraged the hatching of eggs located on the exterior of roots. J2 nematodes were collected and approximately 300,000 were washed ten-times in RNAse-free water using 3,000 g centrifugation. Nematodes were placed in 1 ml 4% resorcinol for 4hrs to induce secretions before pelleting. The supernatant was checked to confirm no nematode contamination before immediate use. Control nematodes were treated with RNAse free water without resorcinol to provide a non-induced sample. Secretions and control samples were spun at 100,000 g for two hours [11,23] to pellet exosomes in a 30 µl fraction.

### Transmission electron microscopy

Exosomes were mounted on 300 mesh, formvar/carbon coated copper grids and allowed to evaporate for 20min. Grids were washed once for 5sec in sterile distilled water and contrasted by uranyl-acetate solution (x 2 drops, 10 sec each). A FEI Tecnai G2 Spirit TEM was used to image exosome samples at 120 kV and 30 k or 150 k magnification.

### Lipid staining

Exosomes were concentrated, as described above. 10 µl of exosomes were added to 10 µl CellMask Orange (5µg/ml) at room temperature for 30 min. 10 µl was analysed via confocal microscopy. The reaction was duplicated with an additional incubation of 5 µl 10% Triton-X100 on ice for 1 hr before reimaging. Control, non-induced solutions were observed similarly.

### Western blotting

Exosomes were concentrated, as described above. Secretions and non-induced samples were mixed with 5 X protein loading buffer (National diagnostics) in a total 20 µl volume, denatured at 9 °C for 5 min and ran on a 12% gradient Mini-PROTEAN gel at a constant voltage of 100 V for 45 min. Proteins were transferred onto nitrocellulose membranes using a wet transfer system at 100 V for 75 min at 4 °C. Membranes were blocked in 5% (w/v) non-fat dry milk and treated with Tris-buffered saline 0.1% Tween-20 (TBST) for 1hr at room temperature. Membranes were incubated overnight at 4°C with anti-CD63 primary antibodies (Abcam; 1:2000 5% milk-TBST). Following three washes in TBST, membranes were incubated with anti-rabbit secondary antibodies (1:10,000 5% milk-TBST) for 1 hr at room temperature. After additional TBST washes, membranes were developed overnight at 4 °C using alkaline phosphatase substrate (SIGMAFAST-BCIP/NBT tablets).

### miRNA extraction, sequencing and annotation

Secretions and non-induced samples from approximately 300,000 resorcinol-induced *M. incognita* J2s were collected, as described above. Nematodes were homogenised post-resorcinol induction. miRNA was extracted from the three treatments by E.Z.N.A MicroRNA Kit (Omega Bio-tek). Fifty ng of miRNA was sent to Genewiz for Small RNA-Seq, returning >20 million reads for each sample. Genewiz provided *de novo* RNA annotations and matches to miRbase libraries of *Caenorhabditis elegans* and *Solanum lycopersicum* (*M. incognita* host). Specifically, raw sequence reads were quality/adapter trimmed (Trimmomatic.v0.30) to retain 18–32 bp sequences and annotated with miRbase.22. Matched sequences were manually confirmed within the nematode genome. For novel microRNA prediction, sequences were aligned to the *M. incognita* genome and subjected to RNA folding/secondary structure analysis (miRDeep2). To identify putative secreted miRNA, data were filtered to remove sequences detected within non-induced samples and with a read count <10. *miR167a* was located within the *M. incognita* genome and life stage expression patterns were determined using WormBase Parasite (PRJEB8714 [24]).

### RNA protection assay

Approximately 300,000 J2s were collected in eight separate batches to provide eight replicates. Exosomes were collected and concentrated as described above. Resorcinol-induced fractions were confirmed to contain exosome-like structures and aliquoted before subject to treatments to determine the robustness of *Mi-miR167a* and its possible enclosure within vesicles. Treatments consisted of, i) control, ii) RNase A (10 µg/ml; 1 hr; 37 °C), iii) Proteinase K (200 µg/ml; 1 hr; 37 °C) followed immediately with the addition of RNase A (10 µg/ml; 1 hr; 37 °C) [25], iv) Triton-X-100 (1%; 1 hr; on ice), v) Triton-X-100 (1%; 1 hr; on ice) followed immediately with the addition of RNase A (10 µg/ml; 1 hr; 37 °C). All samples were subjected to final heat-deactivation of RNase A (10 min; 95 °C). The miRNA were extracted from all samples, as described above, prior to reverse transcription (TaqMan MicroRNA Reverse Transcription Kit, ThermoFisher). Synthesised *Mi-miR167a* were spiked into miRNA depleted samples (treatment v) post-RNA extraction to assess the impact of Triton X-100 treatment on RNA extraction and resultant miRNA detection. *Mi-miR167a* was quantified using a Custom TaqMan Small-RNA Assay using TaqMan Universal PCR Master Mix (ThermoFisher). No-template controls were used to determine background amplification (recovered Ct values of >42). Synthesised *Mi-miR167a* were used to establish a standard curve of miRNA copy number (R ^2^= 0.984, n = 8) from which sample *Mi-miR167a* abundance could be estimated (n = 8).

### *In situ* hybridisation

J2 nematodes were fixed and cut as previously described [26], including an additional 1-ethyl-3-(3-dimethylaminopropyl) carbodiimide (EDC) fixation step to increase miRNA retention [27]. Spatial gene expression profiles were determined by the hybridisation chain reaction (Molecular Instruments Inc, USA) [28].

### Synthetic *Mi-miR167a* treatment

Mature *Mi-miR167a* and its complementary strand were synthesised with a 5’ CY3-tag (Eurofins Genomics) and annealed in 5 X annealing buffer at 90 °C for 1min, decreasing by 0.1 °C/sec to 37 °C and held for 45 min, for a final concentration of 2 µM [22]. The roots of six-day old *Arabidopsis thaliana* were submerged in 0.2 µm dsCY3-Mi-miR167a for 2 hr, before washing three times in water. Mock water treatments were used as negative controls.

Eleven-day old tomato were infected with 100 *M. incognita* J2s in soil-free pouches. 20 nM untagged ds*Mi-miR167a* was prepared as described above and applied to the infected root tips each day for four days. Control plants were treated with 22-nucleotide dsRNA complementary to *gfp*. N = 12. At five days post infection the roots were weighed (no significant difference between treatments) and nematodes were quantified post-acid fuchsin staining. The setup was repeated but with *Mi-miR167a* applications at distant, uninfected root tips to determine systemic effects on root invasion.
